# The first study on the impact of lumpy skin disease outbreaks on monthly milk production on dairy farms in Khon Kaen, Thailand

**DOI:** 10.14202/vetworld.2023.687-692

**Published:** 2023-04-06

**Authors:** Paramintra Vinitchaikul, Veerasak Punyapornwithaya, Suvaluk Seesupa, Sitthinon Phuykhamsingha, Orapun Arjkumpa, Chalutwan Sansamur, Chaiwat Jarassaeng

**Affiliations:** 1 Department of Food Animal Clinic, Faculty of Veterinary Medicine, Chiang Mai University, Chiang Mai, Thailand; 2 Center of Excellence in Veterinary Public Health, Faculty of Veterinary Medicine, Chiang Mai University, Chiang Mai, Thailand; 3 Veterinary Public Health and Food Safety Centre for Asia Pacific, Faculty of Veterinary Medicine, Chiang Mai University, Chiang Mai, Thailand; 4 Division of Theriogenology, Faculty of Veterinary Medicine, Khon Kaen University, Khon Kaen, Thailand; 5 Khon Kaen Dairy Cooperative, Khon Kaen, Thailand; 6 Department of Livestock Development, Animal Health Section, The 4^th^ Regional Livestock Office, Khon Kaen, Thailand; 7 Akkhraratchakumari Veterinary College, Walailak University, Nakhon Si Thammarat, Thailand

**Keywords:** dairy, lumpy skin disease, milk production, outbreaks, Thailand

## Abstract

**Background and Aim:**

Outbreaks of lumpy skin disease (LSD) have resulted in substantial economic losses to the dairy industry in Thailand. This study aimed to determine the influence of LSD outbreaks on monthly milk production levels.

**Materials and Methods:**

Milk production for dairy farms located in Khon Kaen Province, Thailand, belonging to the Khon Kaen Dairy Cooperative, was affected by LSD outbreaks from May to August of 2021. The resulting data were analyzed using general linear mixed models.

**Results:**

It was estimated that the LSD outbreak caused economic losses totaling 2,413,000 Thai Baht (68,943 USD) over the outbreak period. The monthly farm milk production level in May differed from the levels in June and August. Dairy farmers experienced losses between 8.23 and 9.96 tons of milk each month, which equated to between 4180 and 14,440 Thai Baht (119.43 and 412.57 USD) in monthly income.

**Conclusion:**

This study demonstrated that LSD outbreaks on dairy farms resulted in significant farm milk production losses. Our findings will increase awareness among authorities and stakeholders in the dairy industry of Thailand, as well as to assist in the prevention of future LSD outbreaks and minimize the negative impacts of LSD.

## Introduction

Lumpy skin disease (LSD) is caused by the LSD virus belonging to the genus Capripoxvirus within the *Poxviridae* family [1]. LSD is characterized by fever with the appearance of nodular skin lesions covering all parts of the animal’s body and occasionally results in an enlargement of the lymph nodes. Lumpy skin disease is a vector-borne disease, for which the primary route for LSD transmission occurs through certain insect vectors such as stable flies [2–4] and mosquitoes [5, 6]. Lumpy skin disease originated on the African continent and then spread to several countries in Europe, the Middle East, and Asia [7]. At present, LSD outbreaks have been reported in several Asian countries, including Bangladesh [8], China [9], India [10], Bhutan, Nepal [11], Vietnam [12], Myanmar [13], Hong Kong [14], Taiwan [15], Thailand [16], and Malaysia [17], respectively. The first outbreak of LSD in Thailand was discovered in March of 2021 on beef cattle farms located in the northeastern part of the country [16]. Subsequently, LSD outbreaks have been reported in many other Southeast Asia countries [18, 19]. Up until March of 2022, the total number of beef and dairy cattle that have been affected by LSD in the region was 609,073 and 12,317 heads of cattle, respectively, according to the LSD Outbreak Investigation and Control Program instituted by the Department of Livestock Development [20]. During the outbreaks, the infected LSD animals appeared in poor health and their milk production levels were significantly reduced [21].

This disease is a highly contagious and emerging transboundary notifiable viral disease affecting cattle that can have significant economic consequences [2]. These economic impacts not only cause losses in animal productivity but can also drive up the cost of both treatment and vaccination programs. Moreover, restrictions on both live animals and their products must be put in place in response to these outbreaks [2]. LSD can have considerable economic ramifications as the disease has been observed to have a significant negative financial impact on cattle herd owners, customers and the livestock industry [22, 23]. While numerous studies have been published on the effects of LSD epidemics on dairy farms [8, 24, 25], only a few of them have attempted to quantify losses in milk production that occur in the wake of these outbreaks. According to a study conducted in Kenya, milk production decreased from 1.5 to 9.9 L/farm/day [26]. Due to an LSD outbreak, a dairy herd located in Oman reduced milk output by 40%–50% and the loss lasted for several months [27]. The average farm-level losses due to milk yield reductions were 25–100 USD for indigenous breeds and 25–873 USD for exotic cattle breeds, respectively [26].

According to the first nationwide LSD outbreaks in Thailand, one of the most concerning issues discussed among authorities and stakeholders was the negative impact of the outbreaks on milk production on dairy farms. This was because a significant drop in milk production could threaten the financial viability of the country’s dairy farmers. Nonetheless, research on this topic has been very limited. This study aimed to assess the milk production losses caused by LSD outbreaks on dairy farms located in Khon Kaen Province, Thailand.

## Materials and Methods

### Ethical approval

The Khon Kaen Dairy Cooperative provided us with monthly milk production data at the cooperative and farm levels. This study did not include any animal handling or animal-related issues. As a result, animal ethical approval was not required.

### Study period and location

The study was conducted from March to August of 2020 and 2021. The information was gathered from the Khon Kaen Dairy Cooperative, which is located in the Muang and Ban Haet Districts of Khon Kaen Province, Thailand.

### Study area, outbreak farms, and milk production data

In 2021, provincial livestock authorities reported LSD outbreaks on dairy farms located in Muang and Ban Haet Districts, Khon Kaen Province, Thailand. Those dairy farms belong to the Khon Kaen Dairy Cooperative. These dairy farms use a bucket system to collect milk from the herds, and thus quantification of milk production for each cow has been difficult. These dairy farm owners manage and transport raw milk to the dairy cooperative twice each day during morning and evening shifts.

The LSD outbreak lasted 3 months in the dairy cooperative. Lumpy skin disease outbreaks were found in 26, 102, and 5 farms in May, June, and July, respectively, with 531, 1870, and 255 dairy cattle showing clinical signs of LSD. Nonetheless, data on the type of dairy cattle (e.g., heifer, milking, or dry cows) affected by LSD were not available, limiting the ability to determine individual milk loss. As a result, the focus of this study is on farm-level milk production, with the goal of determining farm milk production changes during the outbreak period.

Total monthly cooperative milk production (MCP) data were acquired from all dairy farms and utilized to establish production trends. In addition, monthly farm-level milk production (MFP) data from 129 dairy farms with LSD outbreaks were obtained from the regional dairy cooperative. Data included modified farm identification numbers (farm ID), MFP data and the number of cows milked each month. Thus, true farm ID data and information pertaining to any human issues were not included in this study.

### Statistical analysis

In this study, the relationship of LSD on the reduction in MFP was investigated. We determined whether LSD outbreaks affected MFP from May to August of 2021 using a general linear mixed model (GLMM). This method was chosen because it can be employed to account for the correlation structure of repeated measure data inevitably in the milk production data. Milk production data were repeatedly collected every month over the course of this study period [28]. For the purposes of analysis, the total number of cows milked was included as a covariate factor, while any individual effect that may have occurred to a particular dairy farm was accounted for in the model as a random effect. The most fitted correlation structure for the data was assessed using the Akaike Information Criterion (AIC) [29]. Accordingly, autoregressive 1 had the lowest AIC; thus, the results of the final model were further interpreted based on this correlation structure.

R statistical software (R Foundation for Statistic Computing, Vienna, Austria) and several functions from the “nlme” package [29] were utilized for GLMM analysis. Furthermore, Tukey’s method was employed to make multiple comparisons. The assumptions of GLMM, including homogeneity of variances and normality, were evaluated by examining model residuals and by using fitted values to evaluate the normal Quantile-Quantile plot, respectively [30]. The level of statistical significance was set at p < 0.05. For economic loss calculations, we estimated the standard raw milk price to be 19 Thai Baht per liter or 0.54 US dollars (currency rate at 1 USD equals 35 Thai Baht).

## Results

**Figure-1:**
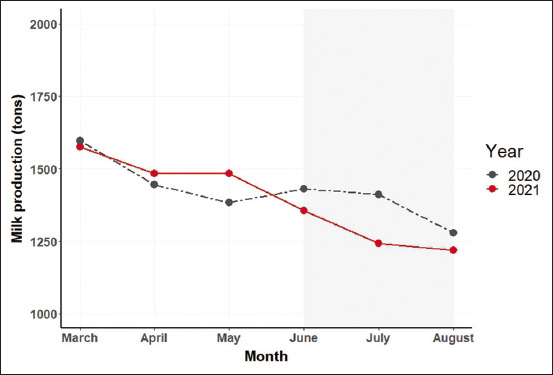
Total monthly cooperative milk production levels from March to August in 2020 and 2021 at a dairy cooperative.

The trends of MCP for farms belonging to the Khon Kaen Dairy Cooperative from March to August in both 2020 and 2021 are presented in Figure-1. It was clearly shown that MCP in 2021 deviated from the pattern established in 2020. Total milk production appeared to be greater in May of 2021 than it was in May 2020. From May to July 2020, MCP seemed to rise, but MCP fell precipitously during the same period in 2021.

Furthermore, MCP declined by 7.55%, 8.21%, and 2.67% from May to June, June to July, and July to August of 2020, respectively. As a result, the dairy cooperative lost money due to a decrease in total milk production per month during the LSD outbreak by 1.84 (0.053), 1.85 (0.053), and 0.55 (0.016) million Thai Baht (USD), respectively, from May to June, June to July, and July to August of 2020 (Table-1). In total, the dairy cooperative’s milk production was diminished by 127 tons during this period. The total milk production price difference was approximately 2,413,000 Thai Baht (68,943 USD).

**Table-1:** Total MCP levels, percentage of milk reduction and economic losses during the LSD outbreak period (May–August, 2021) at a dairy cooperative per month.

Month	MCP (tons)	Percentage in milk reduction (%)	Economic loss in Thai Baht (USD) × 10^6^
May	1,285.02	-	-
June	1,188.04	7.55	1.84 (0.053)
July	1,090.51	8.21	1.85 (0.053)
August	1,061.37	2.67	0.55 (0.016)

MCP=Monthly cooperative milk production, LSD=Lumpy skin disease

Based on data obtained only from LSD outbreak farms from May to August of 2021, there was a decrease in the MFP by 9.96, 9.21, 8.45, and 8.23 tons, respectively. The MFP in August was lower than in May and June. Moreover, in 2021, we estimated that each dairy farmer lost 14,250 (407.14), 14,440 (412.57), and 4180 (119.43) Thai Baht (USD) each month from May to June, June to July, and July to August, respectively (Table-2).

**Table-2:** Monthly farm milk production levels and economic losses during the LSD outbreak period (May–August, 2021) at a smallholder dairy farm per month.

Month	MFP (tons)	Economic loss in Thai Baht (USD)
May	9.96±6.57^a^*	-
June	9.21±5.48^ab^	14,250 (407.14)
July	8.45±4.93^bc^	14,440 (412.57)
August	8.23±4.97^c^	4,180 (119.43)

* Different letters in the same column indicate statistical significance (p *<* 0.05) MFP=Monthly farm milk production, LSD=Lumpy skin disease

## Discussion

In 2021, LSD outbreaks were reported in dairy farms across Thailand. As a result, the negative impacts of the disease have become a major concern among livestock authorities and relevant stakeholders. Estimating the losses due to these disease outbreaks would be of critical interest.

Throughout the outbreak period, the MCP kept declining. The MCP decreased by 7.55% in the 1^st^ month of the outbreak and by 8.21% in the 2^nd^ month. During the outbreak, the MCP continued to fall. The MCP dropped by 7.55% in the 1^st^ month of the outbreak and increased to 8.21% in the 2^nd^ month. These figures appeared to be higher than those calculated at the farm-level, where cows infected with LSD have a mean of 9.9 L decrease in milk yield [26]. For the effects of LSD on MCP, it is necessary to account for multiple factors in addition to animal milk production losses. Monthly cooperative milk production is impacted by the number of LSD outbreak farms belonging to the dairy cooperative, the number of LSD-infected cows on each farm, and the milk yield of each infected cow. For example, the greater the number of LSD-infected cows on the farm, the greater the possibility of a substantial production loss. In addition, the milk production trait is also associated with production losses. For instance, a 5% decrease in milk production for a cow producing 15 L is greater than a 5% decrease for a cow producing 10 L. As shown in a previous study conducted in a similar area to this study, the morbidity rate in LSD outbreak farms is up to 40.5% [21]. Therefore, it was suggested that more than one-third of lactating cows may have reduced their milk production, resulting in a substantial decline in MFP and MCP.

In this study, economic losses due to reductions in milk production at the cooperative level have been estimated to be as high as 2,413,000 Thai Baht (68,943 USD) for this outbreak. The losses due to MCP reduction were greatest in the 2^nd^ month of the outbreak (June) (Table-1). This was likely due to the fact that the peak of outbreaks occurred during this month. In addition, losses were lowest in the last month of the outbreak period (July), which may be attributable to the fact that only a small number of farms had LSD outbreaks during this month, whilst farms that experienced LSD outbreaks in the preceding 2 months may have dairy cattle recovering from the disease and returning to produce more milk than they did during the LSD-infected period.

Furthermore, on average, dairy farmers experienced losses within a range of approximately 4180–14,440 Thai Baht (119.43–412.57 USD) in income due to a reduction of MFP. This estimation was within the range of losses reported in a previous study conducted in Kenya, which reported a loss of between 25 and 873 USD/farm, depending on the breed of cattle, over 70 days due to a drop in milk production [26]. In addition, the estimated economic losses due to reductions in milk production in this study were greater than those estimated in an Ethiopian study, which indicated a 120 USD loss due to diminished milk production [25]. The milk production losses for 2–3 months were comparable with those of previous reports [27, 31, 32]. However, our study was unable to assess the effects of LSD on milk production losses per individual cow. According to an earlier study, the average decrease in dairy milk production per affected animal was 4 L [25]. Furthermore, Ethiopia’s researchers have reported that the economic losses incurred by LSD outbreaks were greater than the losses experienced from food and mouth disease outbreaks on cattle farms [33].

During an LSD outbreak, the majority of cattle on dairy farms can be affected, resulting in a significant drop in milk production. Moreover, the overall state of health of the herd can become worse with decreased appetites, poor body score conditions, anestrous, infertility, incidences of abortion, and increased mortality rates. These findings were consistent with those of earlier studies with regard to the effects of LSD [27, 31]. In terms of opportunity costs, most milking cows were unable to generate the same yield for several months, and in the worst situations, completely dried up due to reduced degrees of milk production. This had a negative influence on the ability of dairy farmers to resume regular production, which was consistent with the outcomes of an earlier study [25]. Furthermore, our research found significant losses in milk production during and after LSD outbreaks. Given that milk output from dairy farms dropped drastically in response to the increasing number of LSD cases over the study period, the great majority of milk production losses were most likely caused by LSD outbreaks rather than by other factors.

The outbreaks have had several negative consequences for dairy farming. Dairy farmers would not only lose income due to reduced bulk milk production, but they would also have to pay for supportive treatment services for their dairy cattle resulting in a considerable rise in expenses. Even though no treatments available for LSD, the best treatment approach would involve supportive therapy. Antibiotics have been extensively used by veterinarians and farmers to prevent secondary infections from skin lesions and pneumonia outbreaks among LSD-infected cattle [34]. Therefore, milk losses did result from the poor states of health of animals infected with LSD, while tainted milk was routinely discarded due to antibiotic withdrawal time periods. There have currently been a limited number of studies on these milk production losses. Farmers must also spend money on LSD vaccinations and pest control measures to avoid future disease outbreaks. However, a recent study has demonstrated a positive net benefit from LSD immunization in herds based on financial analysis, and this practice should be encouraged for ultimate economic effectiveness [25]. Nonetheless, these charges were not reported in the current study due to a lack of access to relevant data.

The scope of this study in terms of economic consequences was limited to direct reductions in milk production levels. There were certain limitations to this study that should be considered when interpreting the findings. First, disorders other than LSD can impair milk production on dairy farms such as mastitis and various infectious diseases. We did not include disease information pertaining to these disorders because relevant data were unavailable. However, as compared to the circumstance in which many dairy cows on most dairy farms exhibit LSD clinical indications and would later lose milk production, this concern about other disorders may have had little impact on monthly milk production rates. Second, because practically all dairy farmers are smallholders who use bucket systems rather than pipeline systems, measuring individual cow milk weight is not a common practice. Therefore, our study was unable to assess the effects of LSD on milk production losses per cow. Finally, the authors would point out limitations in this study were the other confounding factors that can impair milk production and the inability to estimate the effects of LSD on individual milk production losses. For more insight and a better understanding of the economic effects of LSD outbreaks, a follow-up study involving numerous economic variables would be required. Agricultural economists from various sectors will be consulted to learn more about the economic impact of LSD outbreaks.

Regarding the suggestion to reduce economic losses from the LSD outbreak, we recommend a vaccination program in all healthy animals, once a year in dairy cows and in calves at 6 months old if a dam cow was vaccinated prior to parturition, to reduce economic losses caused by LSD outbreaks [20]. Moreover, cattle movements should be strictly regulated during an LSD outbreak [26]. Furthermore, insect vector control programs for mosquitoes and stable flies should be strategic in a dairy farm, especially during the rainy season (July to October) when populations are at their peak [35].

## Conclusion

This is the first study to assess the economic losses due to LSD outbreaks on dairy farms in terms of milk production losses in Thailand’s northeastern region. Economic losses incurred from decreased milk production levels in the dairy cooperative have been anticipated to be as high as 2,413,000 Thai Baht (68,943 USD) or milk production losses of 127 tons. A smallholder dairy farm’s average monthly loss ranged from 4180 to 14,440 Thai Baht (119.43 to 412.57 USD), while milk production losses ranged from 8.23 to 9.96 tons, respectively. The outcomes of this study have provided critical information on the negative impacts of LSD on the economic-based farm-level milk production data obtained from smallholder dairy farms.

## Authors’ Contributions

PV, VP, CJ, SP, and SS: Designed the study. PV, VP, and CJ: Provided oversight for the project. CJ, SP, SS, and OA: Gathered and organized the data from different sources. VP, CJ, SS, CS, and OA analyzed the data and interpreted the results. PV, VP, CJ, SS, and CS: Prepared the initial draft of the manuscript. All authors have read, reviewed, and approved the final manuscript.
